# Schrodinger’s scat: a critical review of the currently available tiger (*Panthera Tigris*) and leopard (*Panthera pardus*) specific primers in India, and a novel leopard specific primer

**DOI:** 10.1186/s12863-016-0344-y

**Published:** 2016-02-09

**Authors:** Pranay Amruth Maroju, Sonu Yadav, Vishnupriya Kolipakam, Shweta Singh, Qamar Qureshi, Yadvendradev Jhala

**Affiliations:** Wildlife Institute of India, Chandrabani, Dehradun, 248001 India

**Keywords:** Species specific primers (SSP), Non-invasive genetic sampling, Fecal DNA, Duplex PCR

## Abstract

**Background:**

Non-invasive sampling has opened avenues for the genetic study of elusive species, which has contributed significantly to their conservation. Where field based identity of non-invasive sample is ambiguous (e.g. carnivore scats), it is essential to establish identity of the species through molecular approaches. A cost effective procedure to ascertain species identity is to use species specific primers (SSP) for PCR amplification and subsequent resolution through agarose gel electrophoresis. However, SSPs if ill designed can often cross amplify non-target sympatric species. Herein we report the problem of cross amplification with currently published SSPs, which have been used in several recent scientific articles on tigers (*Panthera tigris)* and leopards (*Panthera pardus*) in India. Since these papers form pioneering research on which future work will be based, an early rectification is required so as to not propagate this error further.

**Results:**

We conclusively show cross amplification of three of the four SSPs, in sympatric non-target species like tiger SSP amplifying leopard and striped hyena (*Hyaena hyaena*), and leopard SSP amplifying tiger, lion (*Panthera leo persica*) and clouded leopard (*Neofelis nebulosa*), with the same product size. We develop and test a non-cross-amplifying leopard specific primer pair within the mitochondrial cytochrome b region. We also standardize a duplex PCR method to screen tiger and leopard samples simultaneously in one PCR reaction to reduce cost and time.

**Conclusions:**

These findings suggest the importance of an often overlooked preliminary protocol of conclusive identification of species from non-invasive samples. The cross amplification of published primers in conspecifics suggests the need to revisit inferences drawn by earlier work.

**Electronic supplementary material:**

The online version of this article (doi:10.1186/s12863-016-0344-y) contains supplementary material, which is available to authorized users.

## Background

Poaching and habitat loss reduce effective population size and lead to isolation of small populations. These effects combined with genetic drift and inbreeding result in the loss of genetic diversity and threaten to cause local extinctions [1, 2]. Assessment of the genetic diversity and structure of a population helps conservation management in identifying appropriate solutions [3] like restoring connectivity [4, 5], supplementation [6, 7] and reintroductions [8–10]. Advancements in non-invasive genetics have now made it possible to analyse large samples from a variety of sources like hair and faeces, enabling us to have a better insight into the genetic profiles of many endangered and elusive species [11], where otherwise it would be often difficult to obtain information.

The fecal samples of several sympatric species are morphologically similar, rendering field based identification inaccurate [12] and the best way to resolve species identification is through sequencing a portion of mitochondrial gene, which is conserved within a species [13]. However for a large number of samples, the sequencing approach is not cost effective and therefore researchers often rely on species specific primer (SSP)-based Polymerase Chain Reaction and gel-electrophoresis for confirming species identity [14]. Use of species specific primers which amplify only a specific region of highly conserved mitochondrial gene of a particular species can prove handy in screening large scale samples in population studies. However, SSPs are usually designed on a few base pair differences between species [15]. DNA obtained from non-invasive sources are often fragmented [16] and therefore chances of binding with non target species DNA increases with inappropriately designed SSPs.

In our study, we tested the specificity of several often used SSPs [15–17] which were meant to amplify DNA of tigers (*Panthera tigris*) and leopards (*Panthera pardus*) and found two of these [15, 17] to be unreliable. These studies [15, 17], mention that the markers were tested with samples of other sympatric carnivores and herbivores, but no positive amplification was achieved for any species other those the intended. However, our data shows that these markers amplifed not only the target species, but also other non-target sympatric conspecifics. We discuss the seriousness of the implications of our findings on inferences drawn by the published studies that use these markers and emphasize the need to revisit them. We also found that there was no existing reliable leopard specific primer available in literature and for that reason we designed a leopard specific primer and tested its specificity. Subsequently, we selected the most promising tiger specific primer pair (TSP) [16] and used it along with our leopard specific primer (LSP) to develop a duplex PCR approach to simultaneously identify tiger and leopard from fecal DNA, thereby improving the accuracy of species identification from fecal DNA while reducing time and cost.

## Results

### SSPs – specificity assessment

**Fig. 1:**
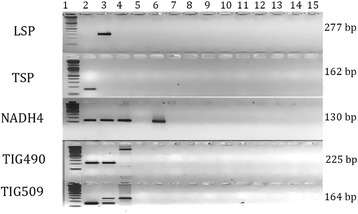
Electrophoresis picture showing amplifciation of various sympatric carnovores and prey (X-axis lanes(with published tiger and leopard specific primers (Y-axis). Lane1-15: 100 bp ladder, tiger, leopard, lion (*Panthera leo persica*), striped hyena (*Hyaena hyaena*), clouded leopard (*Neofelis nebulosa*), caracal (*Caracal caracal*), jungle cat (*Felis chaus*), wolf (*Canis lupus pallipes*), golden jackal (*Canis aureus*), dog (*Canis familiaris*), fox (*Vulpes bengalensis*), sloth bear (*Melursus ursinus*), extraction control, PCR control. Note TIG 509 AND TIG 490, both tiger specific primers amplify leopards and at the same product size. NADH4 a leopard specific primer amplifies tigers, lions and clouded leopards and at the same product size as leopards

On testing the specificity of each available primer pair with the three methods explained, we found that apart from TSP [16] and LSP (designed in this study), all the other primer pairs showed amplification with non-target species (Table 1, Fig. 1) to varying extent. None of these primers showed any amplification with prey species tested

**Table 1:** Results of various specificity tests for tiger and leopard specific primers available in literature

Primer	Target species	In silico PCR positive amplification, Ampliflix		NCBI Primer BLAST match with non-target species	PCR Positive amplification
		Forward	Reverse		
TIG 490^a^	tiger	tiger	Hyena	lion, hyana (striped & spotted), lynx, puma	tiger, leopard, lion
TIG 509^b^	tiger	tiger	tiger, leopard	puma	tiger, leopard, lion
NADH4^c^	leopard	lion, tiger, hyena	lion, leopard	lion	leopard, lion, clouded leopard, tiger
TSP^d^	tiger	tiger	tiger	-	tiger
LSP^e^	leopard	leopard	leopard	-	leopard

^a,b^ – Mukherjee & Mondol et al., 2007 [15]; ^c^– Mondol et al., 2009 [17]; ^d^– Bhagavatula et al.,2006 [16]; ^e^– This study

.

### Leopard specific primer design

The leopard specific primer pair (Additional file 1: Figure S1, Additional file 2: Table S1) designed in this study showed no non-specific annealing and amplified the intended target region with a product size of 277 base pairs (Fig. 1). We found no cross amplification with any of the other species it was tested with (Fig. 1 & Additional file 2: Table S2). With the duplex PCR, the two primer pairs gave sharp amplicons at the expected size of 277 base pairs and 162 base pairs (Additional file 3: Figure S2), confirming the success and accurate amplification of the targeted region in duplex conditions with both reference and fecal samples. The success rate of the duplex was 68 % in fecal samples and 100 % in blood and tissue samples, while the singleplex successfully resolved the species identity for samples where the duplex did not yield any result. Sequenced regions of the LSP amplicon aligned with the expected *P.pardus*. Therefore, any positive amplification observed in DNA extracted from fecal samples corresponds to conclusive species identification between tiger and leopard whose scats were most likely to be confused in field identification.

## Discussion

Currently, several genetic studies on endangered species are based on our ability to get information from minute amounts of DNA from non-invasive sources like hair, scales, faeces, urine and shed skin. Subsequent amplification of microsatellites from this DNA provides useful data for answering questions on conservation and population genetics of these species and populations [18]. However, microsatellites, even those designed for a particular species can cross-amplify in closely related species [19]. Given the ambiguity in distinguishing between different species based on field signs and the propensity of microsatellites to amplify closely related species, determining species identity either by sequencing part of mtDNA or through SSP-PCR based techniques become especially important. Microsatellite based inference on genetic structure, population size, migration and diversity of populations where species identity is potentially confounded due to improperly designed PCR based SSPs are questionable. The duplex reaction halves the cost of the lab analysis, as we can screen for both the species in one reaction. Given that the success rate of the duplex reaction is 66 %, it reduces the cost of analysis significantly, where only the samples that did not yield any result in the duplex reaction could be further tested in a singleplex reaction.

We focussed on distinguishing between tiger and leopard scats, which are often confused due to their morphological similarity [16, 20]. On review of the currently available SSPs for tigers and leopards, we found that three out of the four primers cross-amplify with conspecifics as also reported by Bhavanishankar et al. [21]. Yet, these primers continue to be used in studies [22–24]. Our study provides a confirmation of these findings and further suggests stringent quality checks in designing a species specific primer. As highlighted in the methods section, a combination of in-silico and lab based quality checks are essential for determining the cross-amplification for any primer designed. In all of the reviewed primers, a first in-silico check determined that there was a possibility for cross-amplification of non-target species with these primers. On directly testing it in the lab, we found several other species (lion, striped hyena) that these primers could amplify (Table 1). This becomes especially problematic, when conspecific species, like tiger and leopard amplify and produce a product of the same size (TIG 490, TIG 509, NADH4 – Fig. 1) where, it cannot be differentiated through gel electrophoresis and subsequently forms the basis of further analysis using microsatellites or SNPs.

Given the possibility of cross-amplification, the studies that have used these markers are confounding the allelic richness and diversity between the two species i.e., tigers and leopards. As a consequence, there are several implications for gene flow and population size as both migration rate (m) and effective population size (Ne) are functions of mutation rate and alleleic diversity. For example, the population estimation through camera trapping was pegged at 60 individuals in Kanha Tiger reserve in central India, whereas the DNA based studies estimated it to be 89 individuals [23]. The difference in these numbers could potentially be due to leopard scat being mis-identified as tigers in the first species identification step, as the tiger-specific marker used in this study showed a cross amplification with leopard samples. Mondol et al. [25] estimated the historical tiger population in India, to the south of the Ganges alone to be around 58,200 tigers. Given that they employed an unreliable species specific marker, their conclusions regarding the population size might be inflated and these findings need to be re-visited in light of this evidence.

## Conclusions

We anticipate that the combination of TSP [16] and newly designed LSP primer would give accurate results by correctly identifying species. We caution that though the use of quick cost effective PCR based species identity is relevant and important, it can give erroneous results if proper in-silico and lab based checks are not performed at the SSP design stage. Therefore, it would be prudent to always perform these suggested checks, while using the SSPs for the first time and subsequently test the PCR product through sequencing, to ensure that they are performing as per the design. We also suggest sequencing when a sample is obtained from a new region, before proceeding to rely on PCR based species identity. This would help in avoiding flawed conclusions after elegant analysis due to an oversight at the basic data generation stage. Such oversights can compromise the conservation of endangered species by giving a false sense of security.

## Ethics statement

This paper forms a part of the study undertaken for the country wide assessment of genetic structure of tigers. The research was approved by the technical committee of the National Tiger Conservation Authority as well as by the Training, Research and Academic Council of the Wildlife Institute of India, which also consider ethical aspects of the research. Blood/tissue samples used in the study were obtained with due permission under the Wildlife Protection Act (1972) from the Ministry of Environment, Forest and Climate Change and the Chief Wildlife Wardens of Madhya Pradesh, Rajasthan, and Gujarat.

## Methods

### SSPs – specificity assessment

A total of 4 primer pairs [TSP [16]; TIG 490, TIG 509 [15]; NADH4 [17]] have been widely used to identify species in non invasive genetics studies of tigers and leopards in India. These primers were tested for their specificity using a series of analytical and laboratory based methods to assess their reliability by subjecting a) each of the primer pairs for cross annealing and amplification against complete mitochondrion sequences of sympatric carnivores and prey species by an insilico PCR software AmplifX [26];b) the primer sequences to any non-specific annealing with all vertebrate genomes available in NCBI database using NCBI primer BLAST, an online tool for finding specific primers [27] and c) DNA from reference tissue samples of sympatric carnivores (tiger, leopard, lion (*Panthera leo persica*), striped hyena (*Hyaena hyaena*), clouded leopard (*Neofelis nebulosa*), caracal (*Caracal caracal*), jungle cat (*Felis chaus*), wolf (*Canis lupus pallipes*), golden jackal (*Canis aureus*), dog (*Canis familiaris*), fox (*Vulpes bengalensis*), sloth bear (*Melursus ursinus*)) and prey (pig (*Sus scrofa*), buffalo (*Bubalis bubalis*), cow (*Bos taurus*), goat (*Capra aegagrus hircus*), gaur (*Bos gaurus*), chital (*Axis axis*), sambar (*Rusa Unicolor*) and human (*Homo Sapien*)) to PCR amplification with these primer pairs. DNA from above known reference tissue samples obtained from the forensic repository maintained at the Wildlife Institute of India were isolated using QIAGEN DNeasy Blood & Tissue kit with extraction controls. All extractions and PCRs were conducted in isolated spaces, and to prevent aerosol contamination filter barrier pipette tips were used throughout, along with negative controls for all procedures.

### Leopard specific primer design

We designed a species specific primer pair for identification of leopard from DNA obtained from ambigous non-invasive sources. We aligned the cytochrome b gene of the *Panthera pardus* genome, it’s sympatric carnivores (with which leopard scat could possibly be confused), and potential prey species available in NCBI nucleotide database (see Additional file 2: Table S2) using MEGA V.6 [28]. Variable sites of the cytochrome b region exclusive to leopard genome were manually identified and a primer pair (LSP) was designed based on these sites.

### PCR standardization

LSP was standardized using tissue DNA samples extracted from leopard. We tested for potential cross-amplification by this primer of DNA from other sympatric carnivores and prey species from blood and tissue samples of these individuals. The primer was also tried on DNA obtained from leopard scat (collected from Zoo) and tiger scat (from collared individual) stored in 95 % alcohol. To test the effectiveness of the SSP pair on low quality DNA, we extracted DNA from scats using a modified Guanidinium thiocyanate method [29], with extraction controls. For these PCR reactions, a 12ul reaction volume containing 4ul of 2X MasterMix with HotStart Taq polymerase (Qiagen), 1.2 ul of 2 mg/ml Bovine serum Albumin (BSA), 0.2um unlabelled forward and 0.2um of unlabelled reverse primer, 1ul of Coral Dye (Qiagen) and 5ul of extracted DNA were carried out in Eppendorf thermocycler. PCR conditions were : initial denaturation (95 °C for 15 min), 45 cycles of denaturation (94 °C for 45 s), annealing (Ta for 30 s) and extension (72 °C for 45 s) and a final extension (60 °C for 10–30 min). The Ta for our leopard specific primer was between 51 °C −55 °C with an optimum at 52.5 °C.

To minimize the cost, time and to check for potential cross-contamination of samples from different species during collection and transportation, we developed a method to screen tiger and leopard samples simultaneously with both the leopard specific primer designed in this study and the tiger specific primer pair [16] in one PCR reaction. The primer pairs were standardized for PCR conditions with DNA isolated from leopard and tiger tissue sample on eppendorf Mastercycler Nexus gradient using a duplex PCR protocol. A 15ul duplex PCR reaction volume containing, 6 ul of the Qiagen master mix, 2ul of 2 mg/ml of Bovine Serum Albumin, 0.2 uM of both the forward and reverse of each primer pair, 2ul of coral load dye, and 5ul of the extracted DNA were carried out. PCR conditions were : initial denaturation (95 °C for 15 min), 35 cycles of denaturation (94 °C for 30 s), annealing (Ta of LSP at 52.5 °C - for 30 s) and extension (72 °C for 30 s), followed by 35 cycles of denaturation (94 °C for 30 s), annealing (Ta of TSP at 59 °C - for 30 s) and extension (72 °C for 30 s)and a final extension (60 °C for 10–30 min). PCR negatives and extraction controls were included in each reaction set to monitor contamination. The amplified DNA fragments were analyzed in a 2.5 % agarose gel. We also tested the duplex reactions with scat samples (*n =* 50) and blood and tissue samples (*n =* 8), while simultaneously testing it with tiger and leopard specific primers in a singleplex reaction. To further ensure that the leopard specific primer designed in this study was amplifying the intended target sequence, we sequenced the amplified PCR product (from two reference samples) following gel electrophoresis. The same procedure were followed for DNA obtained from fecal samples of tigers and leopards. The positive amplicons were column purified using QIAquick Gel Extraction Kit, and sequenced on and ABI 3730 automated DNA sequencer. Sequences obtained were subjected to NCBI nucleotide BLAST for confirming species identity.

## Availability of data and materials

All supporting data are included in the manuscript as well as additional files in the supplementary section.

## Additional files

Additional file 1: Figure S1. Sequence alignment of leopard with co-predators and some prey species of the mitochondrial cytochrome b gene used for designing the leopard specific primer. Arrows indicate leopard-specific variation and the region used to design the primers. The forward primer is designed between postions 63 and 85, while the reverse is designed based on leopard specific variation between positions 324 and 339 of the aligned sequences. (JPG 394 kb) Additional file 2: Table S1. Sequence of species specific primer designed for leopards, it’s annealing temperature and product size. **Table S2**: List of species mitochondrial sequences used in alignment for leopard specific primer design, with their accession numbers on NCBI database. (DOCX 76 kb) Additional file 3: Figure S2. Duplex nested PCR of and leopard primer with TSP and LSP. Lane1-6: 100 bp ladder, tiger DNA, leopard DNA, tiger and leopard DNA combined, extraction control, PCR control. (TIF 293 kb)

## Abbreviations

DNA: deoxyribonucleic acid
SSP: species specific primers
PCR: polymerase chain reaction
mtDNA: mitochondrial DNA
TSP: tiger specific primer
LSP: leopard specific primer
SNPs: single nucleotide polymorphisms
Ta: annealing temperature
NCBI: national center for biotechnology information
BLAST: basic local alignment search tool
