# Erratum to: Schrodinger’s scat: a critical review of the currently available tiger (*Panthera Tigris*) and leopard (*Panthera pardus*) specific primers in India, and a novel leopard specific primer

**DOI:** 10.1186/s12863-016-0451-9

**Published:** 2017-03-24

**Authors:** Pranay Amruth Maroju, Sonu Yadav, Vishnupriya Kolipakam, Shweta Singh, Qamar Qureshi, Yadvendradev Jhala

**Affiliations:** Wildlife Institute of India, Chandrabani, Dehradun, 248001 India

## Erratum

Unfortunately, after publication of this article [1], it was noticed that the LSP Forward primer listed in Additional file 2 was incorrect. The corrected supplementary material can be found here.

## Additional file

Additional file 2: Table S1. Sequence of species specific primer designed for leopards, it’s annealing temperature and product size. **Table S2.** List of species mitochondrial sequences used in alignment for leopard specific primer design, with their accession numbers on NCBI database. (DOCX 78 kb)
