# Supplementary figures and images for: Schrodinger’s scat: a critical review of the currently available tiger (Panthera Tigris) and leopard (Panthera pardus) specific primers in India, and a novel leopard specific primer
Source: BMC Genet. 2016 Feb 9;17:37. doi: 10.1186/s12863-016-0344-y (PMC4748499; doi:10.1186/s12863-016-0344-y)

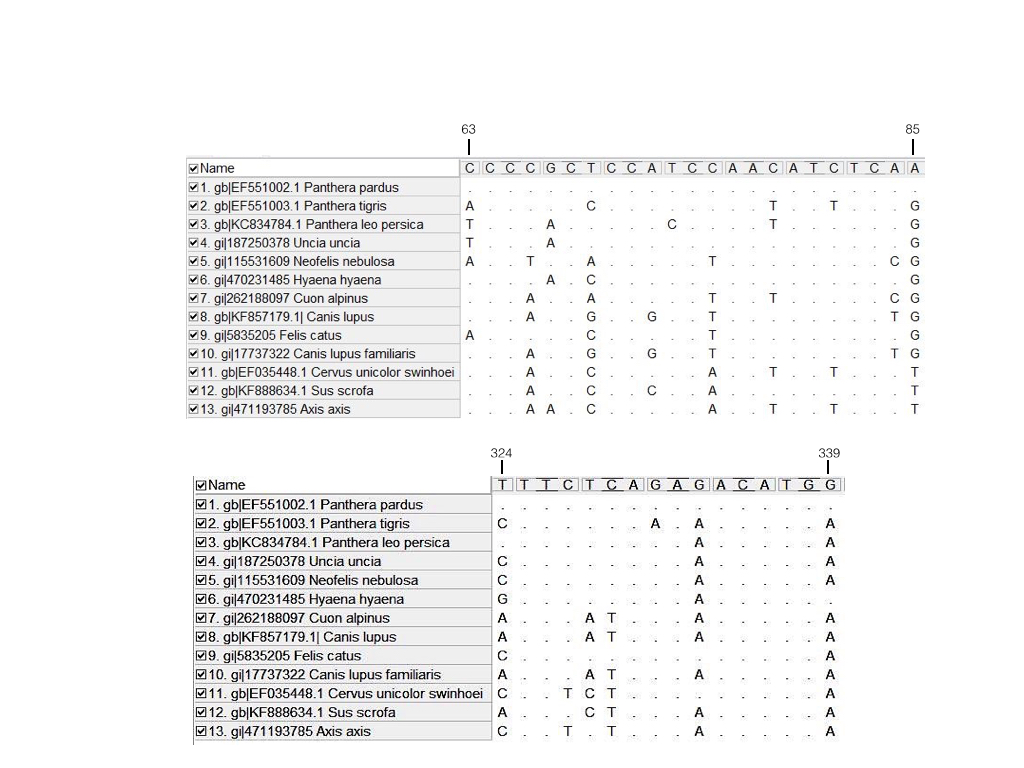

Supplement: Additional file 1: Figure S1. — Sequence alignment of leopard with co-predators and some prey species of the mitochondrial cytochrome b gene used for designing the leopard specific primer. Arrows indicate leopard-specific variation and the region used to design the primers. The forward primer is designed between postions 63 and 85, while the reverse is designed based on leopard specific variation between positions 324 and 339 of the aligned sequences. (JPG 394 kb) [file 12863_2016_344_MOESM1_ESM.jpg]

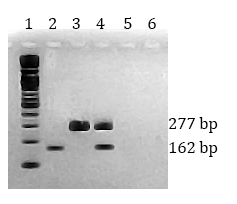

Supplement: Additional file 3: Figure S2. — Duplex nested PCR of and leopard primer with TSP and LSP. Lane1-6: 100 bp ladder, tiger DNA, leopard DNA, tiger and leopard DNA combined, extraction control, PCR control. (TIF 293 kb) [file 12863_2016_344_MOESM3_ESM.tif]
