# Supplementary material for: Schrodinger’s scat: a critical review of the currently available tiger (Panthera Tigris) and leopard (Panthera pardus) specific primers in India, and a novel leopard specific primer
Source: BMC Genet. 2016 Feb 9;17:37. doi: 10.1186/s12863-016-0344-y (PMC4748499; doi:10.1186/s12863-016-0344-y)
Supplement: Additional file 2: Table S1. — Sequence of species specific primer designed for leopards, it’s annealing temperature and product size. Table S2: List of species mitochondrial sequences used in alignment for leopard specific primer design, with their accession numbers on NCBI database. (DOCX 76 kb) [file 12863_2016_344_MOESM2_ESM.docx]

Table S1 : Sequence of species specific primer designed for leopards, it’s annealing temperature and product size

| Primer | Sequence | Annealing Temperature | Amplicon Size (in bp) |
| --- | --- | --- | --- |
| LSP Forward | 5'-ATAAAAAATCAGGAATGGTG-3' | 52.5°C | 277 |
| LSP Reverse | 5'-CCATGTCTCTGAGAAA-3' |  |  |

Table S2: List of species mitochondrial sequences used in alignment for leopard specific primer design, with their accession numbers on NCBI database

| S.No | Species | Accession number |
| --- | --- | --- |
| 1 | Panthera tigris tigris | KF892541 |
| 2 | Panthera pardus | EF551002 |
| 3 | Panthera leo persica | KF776494 |
| 4 | Acinonyx jubatus | NC_005212 |
| 5 | Neofelis nebulosa | NC_008450 |
| 6 | Felis catus | NC_001700 |
| 7 | Hyena hyena | NC_020669 |
| 8 | Cuon alpinus | NC_013445 |
| 9 | Capra aegagrus hircus | KF952601 |
| 10 | Sus scrofa | KF888634 |
| 11 | Canis lupus familiaris | U96639 |
| 12 | Axis axis | NC_020680 |
| 13 | Melursus ursinus | NC_009970 |
| 14 | Capra aegagrus hircus | KF952601 |
| 15 | Canis aureus | AY291433 |
| 16 | Bos gaurus | NC_024818 |
| 17 | Homo sapiens (Cambridge reference sequece) | NC_012920 |
