# Supplementary material for: Erratum to: Schrodinger’s scat: a critical review of the currently available tiger (Panthera Tigris) and leopard (Panthera pardus) specific primers in India, and a novel leopard specific primer
Source: BMC Genet. 2017 Mar 24;18:28. doi: 10.1186/s12863-016-0451-9 (PMC5366152; doi:10.1186/s12863-016-0451-9)
Supplement: Additional file 2: Table S1. — Sequence of species specific primer designed for leopards, it’s annealing temperature and product size. Table S2. List of species mitochondrial sequences used in alignment for leopard specific primer design, with their accession numbers on NCBI database. (DOCX 78 kb) [file 12863_2016_451_MOESM1_ESM.docx]

Table S1 : Sequence of species specific primer designed for leopards, it’s annealing temperature and product size

| Primer | Sequence | Annealing Temperature | Amplicon Size (in bp) |
| --- | --- | --- | --- |
| LSP Forward | 5'-TCCCCGCTCCATCCAACATCTCAA C -3' | 52.5°C | 277 |
| LSP Reverse | 5'-CCATGTCTCTGAGAAA-3' |  |  |

Table S2: List of species mitochondrial sequences used in alignment for leopard specific primer design, with their accession numbers on NCBI database

| S.No | Species | Accession number |
| --- | --- | --- |
| 1 | Panthera tigris tigris | KF892541 |
| 2 | Panthera pardus | EF551002 |
| 3 | Panthera leo persica | KF776494 |
| 4 | Acinonyx jubatus | NC_005212 |
| 5 | Neofelis nebulosa | NC_008450 |
| 6 | Felis catus | NC_001700 |
| 7 | Hyena hyena | NC_020669 |
| 8 | Cuon alpinus | NC_013445 |
| 9 | Capra aegagrus hircus | KF952601 |
| 10 | Sus scrofa | KF888634 |
| 11 | Canis lupus familiaris | U96639 |
| 12 | Axis axis | NC_020680 |
| 13 | Melursus ursinus | NC_009970 |
| 14 | Capra aegagrus hircus | KF952601 |
| 15 | Canis aureus | AY291433 |
| 16 | Bos gaurus | NC_024818 |
| 17 | Homo sapiens (Cambridge reference sequece) | NC_012920 |
